# Generation of Pure Green Up-Conversion Luminescence in Er^3+^ Doped and Yb^3+^-Er^3+^ Co-Doped YVO_4_ Nanomaterials under 785 and 975 nm Excitation

**DOI:** 10.3390/nano12050799

**Published:** 2022-02-26

**Authors:** Natalia Stopikowska, Marcin Runowski, Przemysław Woźny, Stefan Lis, Peng Du

**Affiliations:** 1 Department of Rare Earths, Faculty of Chemistry, Adam Mickiewicz University, Uniwersytetu Poznańskiego 8, 61-614 Poznań, Poland; natalia.stopikowska@amu.edu.pl (N.S.); przemyslaw.wozny@amu.edu.pl (P.W.); blis@amu.edu.pl (S.L.); 2 Departamento de Física, Universidad de La Laguna, Apartado de Correos 456, E-38200 San Cristóbal de La Laguna, Spain; 3 Department of Microelectronic Science and Engineering, School of Physical Science and Technology, Ningbo University, Ningbo 315211, China

**Keywords:** lanthanide ions, up-conversion, nanomaterials, rare earth ions, pure green luminescence

## Abstract

Materials that generate pure, single-color emission are desirable in the development and manufacturing of modern optoelectronic devices. This work shows the possibility of generating pure, green up-conversion luminescence upon the excitation of Er^3+^-doped nanomaterials with a 785 nm NIR laser. The up-converting inorganic nanoluminophores YVO_4_: Er^3+^ and YVO_4_: Yb^3+^ and Er^3+^ were obtained using a hydrothermal method and subsequent calcination. The synthesized vanadate nanomaterials had a tetragonal structure and crystallized in the form of nearly spherical nanoparticles. Up-conversion emission spectra of the nanomaterials were measured using laser light sources with λ_ex_ = 785 and 975 nm. Importantly, under the influence of the mentioned laser irradiation, the as-prepared samples exhibited bright green up-conversion luminescence that was visible to the naked eye. Depending on the dopant ions used and the selected excitation wavelengths, two (green) or three (green and red) bands originating from erbium ions appeared in the emission spectra. In this way, by changing the UC mechanisms, pure green luminescence of the material can be obtained. The proposed strategy, in combination with various single-doped UC nanomaterials activated with Er^3+^, might be beneficial for modern optoelectronics, such as light-emitting diodes with a rich color gamut for back-light display applications.

## 1. Introduction

Up-conversion (UC), anti-Stokes luminescence is a phenomenon where two or more low-energy photons are converted into one photon of higher energy. In this way, the accumulated energy in the system, usually absorbed by the sensitizer ions, is transferred to the activator ions [1,2,3,4,5]. In order to generate efficient UC luminescence, the inorganic matrices co-doped with rare earth ions (typically Ho^3+^, Er^3+^ and Tm^3+^) are commonly used, such as vanadates, phosphates, borates, fluorides, oxides etc. [1,2,3,6,7,8,9,10,11,12,13,14,15,16]. Currently, up-converting (nano)materials are commonly studied in terms of the components of solar cells [17], bioimaging [18,19], nanothemrometry [3,20,21,22,23,24,25,26], forensics [27], composite membranes etc. [28].

Rare-earth vanadates are a group of compounds that are used in many fields of science due to their favorable physicochemical properties, such as chemical stability, relatively low phonon energy (≈900 cm^−1^) [29], lower cytotoxicity than quantum dots [30], simple and eco-friendly synthesis method [31,32,33,34] and so forth.

Importantly, the vanadate-based materials and nanomaterials have much better thermal stability (even above ≈1300 K), in contrast to their fluoride analogues (commonly studied luminescent micron-sized and nano-sized particles), which begin to oxidize and decompose usually above ≈700 K [10,15,25,26,27,29]. 

Moreover, due to the doping of lanthanide ions (Ln^3+^) in their internal structure, such materials are optically active, and may exhibit luminescence phenomena upon appropriate photoexcitation, showing characteristic, sharp and narrow emission bands, corresponding to the Ln^3+^ 4f-4f transitions [20,35]. The vanadate matrices are often used in the conventional luminescence research and also in UC emission studies. In the first case of generating UV-excited luminescence, the emitter ions are, e.g., Eu^3+^, Pr^3+^, Sm^3+^, Dy^3+^, Ho^3+^ and Nd^3+^ [36,37,38,39,40,41,42,43,44]. 

In the case of generation of the UC emission, the most frequently used dopant ions are the following systems: Yb^3+^-Er^3+^, Yb^3+^-Tm^3+^ and Yb^3+^-Ho^3+^, [45,46,47,48]; however, triple or even four-fold doped systems are also known, such as Yb^3+^-Ho^3+^-Nd^3+^ and Yb^3+^-Tm^3+^-Ho^3+^-Er^3+^ [49,50]. One of the most commonly studied materials is YVO_4_ doped with Yb^3+^-Er^3+^. In the case of this system, scientists typically use a conventional NIR laser emitting wavelength of 975–980 nm to generate UC emission [27,31,51,52,53,54,55,56]. 

Woźny et al. reported the UC emission spectra for the YVO_4_: Yb^3+^-Er^3+^ obtained using the co-precipitation method, without the calcination process. In this case, the average size of the material was estimated at 21 nm, and an intense red emission band of Er^3+^ (^4^F_9/2_⟶^4^I_15/2_ transition) located around 650 nm was observed in the emission spectrum (λ_ex_ = 975 nm) [57]. Szczeszak et al. obtained an analogous material using the Pechini method [58]. The material showed high agglomeration, and the average grain size was estimated at 20–50 nm. In that case, the same red emission band originating from Er^3+^ was also observed (λ_ex_ = 980 nm). 

Meng et al. obtained YVO_4_: Yb^3+^-Er^3+^ material using the co-precipitation method [51]. They focused on synthesizing the vanadate materials with different grain sizes, i.e., particles with average sizes of 20 and 60 nm, as well as 1 µm. The research showed the effect of the material size on the intensity of the band coming from the mentioned Er^3+^ transition, i.e., ^4^F_9/2_⟶^4^I_15/2_. In this case, the intensity of the red band decreased with the increase of the particle size. 

However, there are also reports about YVO_4_: Yb^3+^-Er^3+^ materials generating conventional (Stokes) emission, such as the contribution of T. Tsuboi, who investigated the absorption and emission characteristics of this material in the spectral ranges of 200–2000 and 400–1750 nm, respectively, using 671, 355 and 266 nm excitation wavelengths. This author observed not only the emission bands corresponding to Er^3+^ but also the emission band related to Yb^3+^ emission [57].

The discussed materials, based on vanadate matrices are also thermally very stable, [59,60,61], and therefore they are used as thermochromic phosphors, scintillators or optically active components of lasers [62] as well as contact-less temperature sensors [63,64], optical high pressure and vacuum sensors [29] and for fingerprint detection [27]. 

From the point of view of the use of nanomaterials in electronics and optoelectronics, their synthesis has many advantages, such as the possibility of preparation in large quantities with the desired composition, size, shape and structure reproducibility etc. During the preparation of vanadate, a water-based system is used, which provides a number of advantages, such as simplicity, safety, convenience, ease of transfer to large-scale production and no harmful organic solvents, which is very important from the point of view of green chemistry [31,62,65,66].

Here, we present the possibility of generating bright, green UC luminescence, from nanomaterials based on yttrium vanadate matrices doped with Er^3+^ or Yb^3+^-Er^3+^ ions. The optically active compounds were obtained using a hydrothermal method and post-treatment calcination. The nanomaterials synthesized showed pure green up-conversion luminescence, which was clearly visible to the naked eye, under continuous laser excitations, alike at 785 nm (not widely used thus far) and 975 nm. 

Importantly, in the case of 785 nm excitation, the emission spectrum of the single-doped nanomaterial (YVO_4_: Er^3+^) does not have any contribution of the red emission band of Er^3+^ (^4^F_9/2_⟶^4^I_15/2_ transition). Our studies show that the excitation wavelength and the presence of sensitizer ions play important roles in achieving pure green UC emission in vanadate nanomaterials. 

These results and the developed strategy may be particularly important from the point of view of electronics and materials engineering, not only in utilizing vanadates but also other Er^3+^-doped UC nanomaterials (e.g., molybdates and tungstates) excited at higher-energy NIR lasers. This is because the possibility of generating pure-color emission can be used for the manufacturing of modern optoelectronics, new light sources, optically active components of various devices etc.

## 2. Materials and Methods

### 2.1. Materials

RE_2_O_3_ (RE = Y^3+^, Yb^3+^ and Er^3+^) purchased from Stanford Materials (Stanford, CA, USA; 99.99%), were dissolved separately in 35–38% HCl from POCh. S.A. (Gliwice, Poland) to synthesize the corresponding RECl_3_. Ammonium metavanadate (ACS reagent; ≥99.0%) and PEG 6000 were purchased from Sigma Aldrich (Darmstadt, Germany). Sodium hydroxide was purchased from POCh. S.A. (Gliwice, Poland, pure p.a.; 98.8%). Deionized water was used in each experiment.

We detail the synthesis of (A) YVO_4_: 2% Er^3+^ and (B) YVO_4_: 20% Yb^3+^ and 2% Er^3+^. To synthesize 0.25 g of a given nanomaterial, the aqueous solutions of YCl_3_ and ErCl_3_ were mixed together in a molar ratio 0.98:0.02, i.e., 2.362 mL of 0.5 M YCl_3_ and 0.048 mL of 0.5 M ErCl_3_ for the product (A); and the aqueous solutions of YCl_3_, YbCl_3_ and ErCl_3_ were mixed in a molar ratio 0.78:0.2:0.02, i.e., 1.739 mL of 0.5 M YCl_3_, 0.446 mL of 0.5 M YbCl_3_ and 0.045 mL of 0.5 M ErCl_3_ for the product (B). Subsequently, 10 mL of water was added to the solutions of Ln^3+^ ions. Next, 0.25 g of PEG 6000 (anti-agglomeration agent) was added and dissolved in each of the as-prepared solutions.

The solutions containing vanadate ions were prepared by dissolving 0.141 g of NH_4_VO_3_ in 20 mL of water for the product (A); and 0.130 g of NH_4_VO_3_ for the product (B). An aqueous sodium hydroxide solution (15 mL) was added to each solution of ammonium metavanadate, at a molar ratio of 1:1. The solutions containing vanadate ions were heated up to 343 K to obtain transparent aqueous solutions and then added dropwise to the continuously stirred solutions of Ln^3+^.

Then, water (up to 40 mL) was added to the as-prepared solutions, and, in the next step, the pH of each solution was adjusted to ≈ 10, using a 1.5 M solution of NaOH. The obtained mixtures were then transferred into Teflon-lined vessels and hydrothermally treated in an autoclave (for 18 h at 453 K).

Afterwards, the obtained white precipitates were dispersed several times in ethanol and water and centrifuged to purify the final products. The obtained products, i.e., YVO_4_: Er^3+^ and YVO_4_: Yb^3+^ and Er^3+^ were dried in an oven at 358 K for 15 h. Finally, the samples were ground in an agate mortar. Later, in order to enhance the crystallinity and the luminescence signal intensity of the products, they were calcined in a furnace for 4 h at 1173 K. After calcination, the products were ground again in an agate mortar.

### 2.2. Characterization

Powder X-ray diffraction patterns (XRD) were measured using a Bruker AXS D8 Advance diffractometer (Billerica, MA, USA) in Bragg–Brentano geometry (Cu Kα radiation λ = 0.15406 nm). Transmission electron microscopy (TEM) (Hitachi HT7700, Ltd. Tokyo, Japan) images were taken with a Hitachi HT7700 transmission electron microscope (100 kV accelerating voltage). An Andor Shamrock 500i spectrometer (Andor Technology Ltd., Belfast, UK), coupled with a silicon iDus CCD camera, working as a detector, was used for the emission spectra measurements. The samples were excited by the use of the fiber-coupled, solid-state diode pumped (SSDP) 975 and 785 nm lasers, i.e., FC-975-2W (CNI; Changchun, China) and LW-785-120-C12-DM (Lambdawave, Wrocław, Poland), respectively.

In both cases, the beam spot sizes were ≈200 µm (Gauss profile), and the laser power was adjusted to ≈100 mW, for both excitation wavelengths, which corresponds to the power densities of ≈50 W cm^−2^. The luminescence decay curves were recorded using a 200 MHz Tektronix MDO3022 oscilloscope, coupled to the R928 PMT (Hamamatsu, Shimokanzo, Japan) and a QuantaMaster™ 40 spectrophotometer (Photon Technology International, Birmingham Rd, Birmingham UK). A tunable Opolette 355LD UVDM, nano-second pulsed laser, with a repetition rate of 20 Hz (Opotek Inc., Faraday Ave Suite E, Carlsbad, CA, USA), was used as the excitation source.

## 3. Results

### 3.1. Structure and Morphology

**Figure 1:**
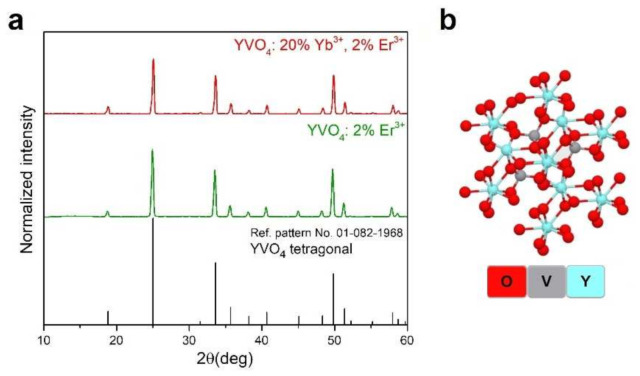
(**a**) Powder XRD patterns of the obtained YVO_4_: 2% Er^3+^ and YVO_4_: 20% Yb^3+^ and 2% Er^3+^ compounds and (**b**) a graphical representation of the arrangement of atoms in the crystal lattice of YVO_4_.

The recorded XRD patterns of the obtained nanomaterials: YVO_4_: 2% Er^3+^ and YVO_4_: 20% Yb^3+^ and 2% Er^3+^ (Figure 1a) agree with the reference pattern from the ICDD database (International Centre for Diffraction Data, card no. 01-082-1968) of the tetragonal YVO_4_, crystallizing in the *I41/amd* space group. Due to the nanocrystallinity of the particles obtained, a significant broadening of reflexes was observed.

**Figure 2:**
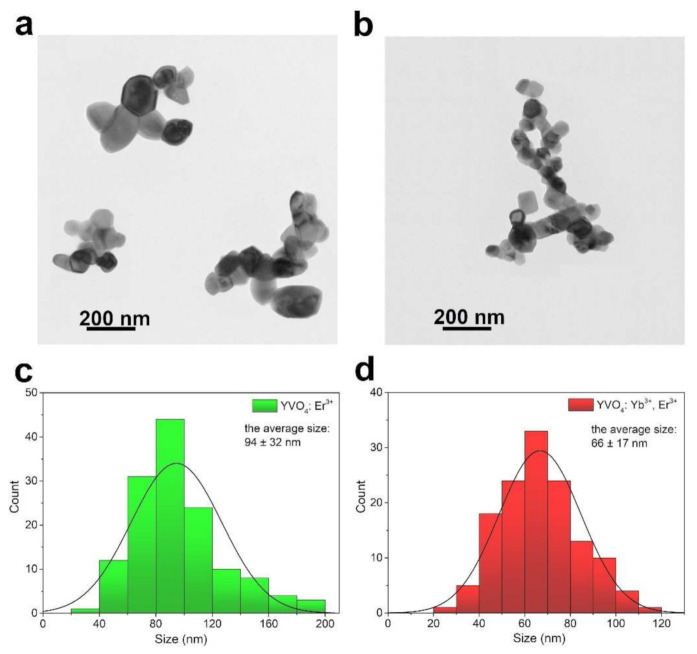
(**a**,**b**) TEM images and (**c**,**d**) corresponding size distribution histograms of the obtained nanomaterials, i.e., (**a**,**c**) YVO_4_: Er^3+^ and (**b**,**d**) YVO_4_: Yb^3+^ and Er^3+^.

Figure 1b shows a graphical representation of the arrangement of atoms in the synthesized crystal structures. In the cases of the Er^3+^ doped and Yb^3+^-Er^3+^ co-doped YVO_4_ materials, the Y^3+^ ions in the crystal lattice were partly substituted either by Er^3+^ ions or by Yb^3+^ and Er^3+^ ions, respectively. TEM images (Figure 2a,b) show that the obtained compounds were composed of irregular, agglomerated nanoparticles (NPs), and their average sizes were around 94 ± 32 nm for YVO_4_: Er^3+^ (Figure 2c) and 66 ± 17 nm for YVO_4_: Yb^3+^ and Er^3+^ (Figure 2d).

### 3.2. Luminescence Properties

**Figure 3:**
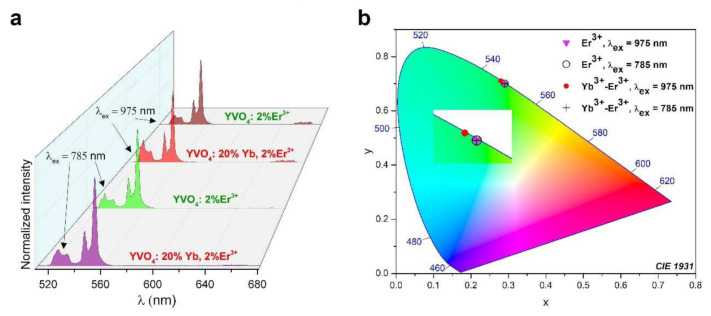
(**a**) Normalized UC emission spectra of the synthesized YVO_4_: Er^3+^ and YVO_4_: Yb^3+^ and Er^3+^ nanomaterials, measured at two different laser excitations (λ_ex_ = 785 or 975 nm; ≈50 W/cm^2^) and (**b**) the corresponding chromaticity diagram (CIE 1931).

The doping concentrations (20 mol.% of Yb^3+^ and 2 mol.% of Er^3+^) were chosen based on our previous studies as well as the literature data [27,55,56]. This dopant ratio provides optimal, intense UC emissions, due to the efficient energy transfer process between Yb^3+^ and Er^3+^. UC emission spectra were recorded in the range of 500–680 nm (Figure 3a; λ_ex_ = 785 or 975 nm; pump power density ≈ 50 W/cm^2^). The synthesized nanomaterials exhibit a very bright green UC luminescence that is clearly visible to the naked eye.

When using λ_ex_ = 785 nm, the emission spectrum of YVO_4_: Er^3+^ consists of only two narrow, sharp bands from Er^3+^: ^2^H_11/2_⟶^4^I_15/2_ (530 nm) and ^4^S_3/2_⟶^4^I_15/2_ (550 nm), associated with its *4f−4f* radiative transitions (both located in the green region of the spectrum). However, in the case of using λ_ex_ = 975 nm, the YVO_4_: Er^3+^ and YVO_4_: Yb^3+^ and Er^3+^ compounds exhibit an additional low intense band located around 670 nm, corresponding to the ^4^F_9/2_⟶^4^I_15/2_ transition of Er^3+^. During the excitation of the co-doped nanomaterial YVO_4_: Yb^3+^ and Er^3+^ with a 785 nm laser, a low-intensity band located around 670 nm was also visible. All bands are split into several Stark components due to the effects of the crystal-field.

In both synthesized nanomaterials YVO_4_: Er^3+^ and YVO_4_: Yb^3+^-Er^3+^, a pure green color of luminescence was achieved using λ_ex_ = 785 nm, as well as for the single-doped YVO_4_:Er^3+^ (without ytterbium co-doping) excited at 975 nm, as presented in the chromaticity diagram in Figure 3b. However, for the nanomaterial YVO_4_: Yb^3+^-Er^3+^ excited at 975 nm, the resulting color coordinates are slightly shifted (see Figure 3b).

Based on the measured UC emission spectra, we determined the values of color coordinates, summarized in Table 1, where they are all on the edge of green region, and this indicates a higher color purity of the resulting emissions originating from the obtained nanomaterials. To confirm this deduction, we estimated the color purity utilizing the following equation [67,68]:

(1) $$\mathrm{Color}\;\mathrm{purity}=\frac{\sqrt{{\left(x-x_{i}\right)}^{2}+{\left(y-y_{i}\right)}^{2}}}{\sqrt{{\left(x_{d}-x_{i}\right)}^{2}+{\left(y_{d}-y_{i}\right)}^{2}}}\times 100\%$$

where (*x*,*y*) denote the color coordinates of the developed nanoluminophores; (*x*_i_,*y*_i_) are the color coordinates of the white illuminate point, which have fixed values of (0.3101,0.3162) [69], and (*x_d_*,*y_d_*) are the color coordinates of the dominated emissions, whose values can be determined through extending the straight line between the points of (*x*,*y*) and (*x_i_*,*y_i_*) to the other side (edge of the CIE diagram) [70]. The determined values of (*x_d_*,*y_d_*) and color purities for the studied nanomaterials are summarized in Table 1.

As expected, the single-doped sample (YVO_4_:Er^3+^) excited at 785 nm reveals superior color purity of its green emission, namely, 98.9%. The color purity values change slightly with the manipulation of the chemical composition of the materials (dopant ions) and the excitation wavelengths. In addition to the color coordinates and color purity, the correlated color temperature (CCT) also plays an important role in determining the color properties of the generated emissions, and its value can be calculated using the following equations [70]:

(2) $$\mathrm{CCT}=-437n^{3}+3601n^{2}-6846n+5514.31$$

(3) $$n=\;\left(x-x_{e}\right)/\left(y-y_{e}\right)$$

where (*x_e_*,*y_e_*) have fixed values of (0.3320,0.1858). Thereby, via using these aforementioned equations, the CCT values for the emissions of Er^3+^-doped and Er^3+^/Yb^3+^-codoped YVO_4_ nanomaterials excited with different wavelengths (785 or 975 nm) are calculated and presented in Table 1. As disclosed, by changing the dopant content and excitation wavelength, CCT values vary in the range of 6018 to 6334 K.

Additionally, it is worth noting that the intensity of the red emission band of Er^3+^ (^4^F_9/2_⟶^4^I_15/2_) may also be influenced by the synthesis method of the luminescent material [51,52,55,71,72]. In general, based on the available literature data, it can be concluded that thermal treatment, i.e., high temperatures used during the solid-state method or post-synthesis calcination favor green emissions—namely, the relative intensity of the red emission band is significantly lower compared to the green emission bands [51,52,55,71,72].

**Figure 4:**
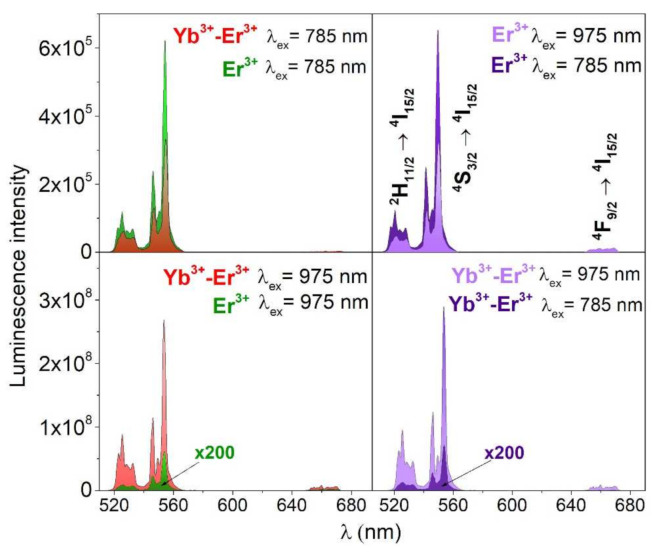
Non-normalized UC emission spectra of the obtained nanomaterials YVO_4_: Er^3+^ and YVO_4_: Yb^3+^-Er^3+^; λ_ex_ = 785 or 975 nm (≈50 W/cm^2^).

Figure 4 shows how the selected excitation wavelengths and the elemental composition (single- or co-doped samples) affect the intensity of UC emission of the nanomaterials studied. The excitation of the samples at 785 nm, resulted in higher UC emission intensity for the single-doped YVO_4_:Er^3+^ compound. This is most plausibly because, in the case of a co-doped material, after the excited state absorption of Er^3+^, some of the excitation energy is transferred back to the Yb^3+^ ions, namely, via Er^3+^→Yb^3+^ back energy transfer (BET), which may further relax non-radiatively and radiatively (NIR emission of Yb^3+^), resulting in a decrease in the intensity of Er^3+^ UC emission.

While, as expected, the most intense UC luminescence is shown by the material co-doped with Yb^3+^-Er^3+^ ions, excited at 975 nm—namely, two orders of magnitude higher compared to the second excitation wavelength and the single-doped sample (see Figure 4). This is due to the high absorption cross-section of Yb^3+^ in the NIR range, centered around 975 nm (^2^F_7/2_⟶^2^F_5/2_ transitions of Yb^3+^) and the effective energy transfer UC (ETU) from the sensitizing Yb^3+^ ions to the emitting Er^3+^ ions (Yb^3+^⟶Er^3+^ ETU).

Importantly, using the 785 nm laser (allowing generation of the pure green luminescence), it is possible to excite the samples in the range of the first biological window (I-BW), i.e., 650–950 nm [3,7,13]. In this range, the disturbing factors, including scattering or/and absorption of the laser beam by the biological tissues, are less pronounced, allowing for better penetration of the tissue by the excitation beam. This feature is particularly important, e.g., in the field of development of optical contactless nano-thermometers, which are particularly useful in biological and medical research and applications [7,13,20].

According to the available literature data, the up-converting materials obtained thus far, based on the Er^3+^ doped or Yb^3+^-Er^3+^ co-doped inorganic compounds do not show pure, green emission (without the influence of the red emission band) under the NIR laser excitation, i.e., in the I-BW spectral range. Table 2 summarizes the spectral characteristics of the luminescent nanomaterials based on the Er^3+^ emission in the vanadate matrices, among which, YVO_4_ is the most commonly used host.

To date, pure green emission could be achieved only in the case of the conventional, UV-excited, down-shifting phosphors. Whereas, in the case of the up-converting materials, excited either in the I-BW or beyond, the red band was inherently present in the emission spectra.

**Figure 5:**
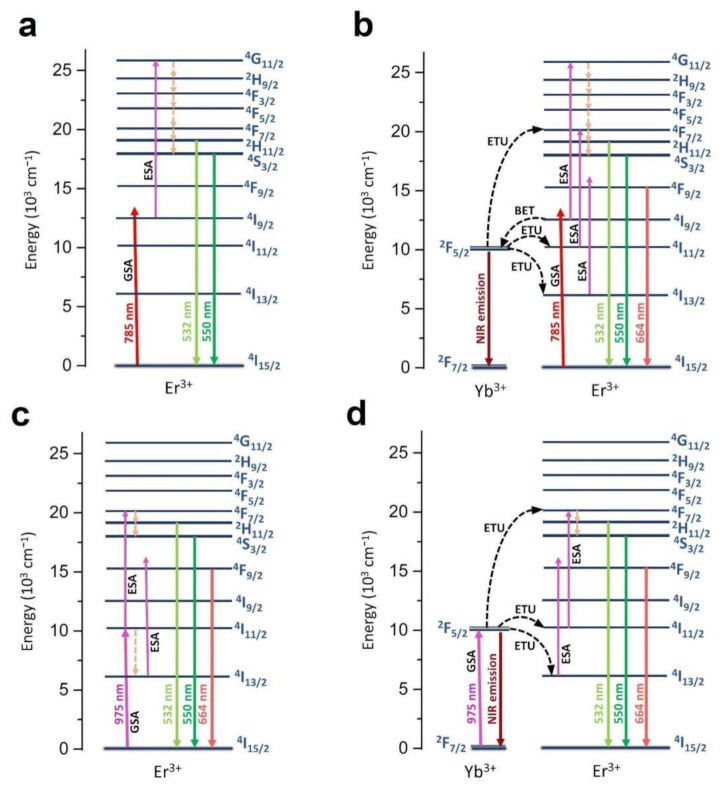
Energy level diagrams showing the main radiative (continuous lines) and non-radiative (dashed lines) processes occurring in the nanomaterials studied, i.e., (**a**) YVO_4_: Er^3+^, λ_ex_ = 785 nm; (**b**) YVO_4_: Yb^3+^-Er^3+^, λ_ex_ = 785 nm; (**c**) YVO_4_: Er^3+^, λ_ex_ = 975 nm; (**d**) YVO_4_: Yb^3+^-Er^3+^, λ_ex_ = 975 nm.

The main radiative and non-radiative processes occurring in the studied nanomaterials are shown in Figure 5. In the case of the commonly studied systems, which are the Yb^3+^-Er^3+^ co-doped UC phosphor excited at 975 nm, the already discussed and well-established ETU mechanism dominates (Figure 5d) [2,84,85,86,87,88]. On the other hand, for the single-doped materials (a,c), co-doped materials (b) excited at 785 nm, the mechanisms responsible for UC emission of Er^3+^ are predominantly ground state absorption (GSA) and excited state absorption (ESA) processes.

In the latter case (b), the ETU mechanism may also contribute; however, it appears to be less efficient compared to the GSA and ESA mechanisms. Importantly, pure green luminescence, i.e., no red emission band in the spectrum, can only be fully achieved for a single-doped nanomaterial excited at 785 nm (directly into the ^4^I_9/2_ level of Er^3+^). This is because, only in that case (a), the ^4^F_9/2_ level cannot be effectively populated (at least at room temperature), as evidenced by the measured spectra and energy level diagrams depicted.

The only way to theoretically populate this level would be a multi-phonon relaxation (from the ^4^S_3/2_ level), which is often considered in the literature to be responsible for the red emission of Er^3+^ [2,85,86]. However, as the ^4^S_3/2_ and ^4^F_9/2_ levels are separated by ≈3000 cm^−1^, at least four phonons are required to populate the lower-lying state (assuming the highest-energy phonon mode is ≈ 900 cm^−1^ in the vanadate crystal lattice), which evidently makes the mentioned multi-phonon relaxation process less efficient, compared to the competing green emission from the ^4^S_3/2_ state in the system studied. Whereas, in other cases (b–d), a red emission band can be observed because the ^4^F_9/2_ level can be populated via the ESA mechanism from the ^4^I_13/2_ level (see the energy level diagrams in Figure 5).

**Figure 6:**
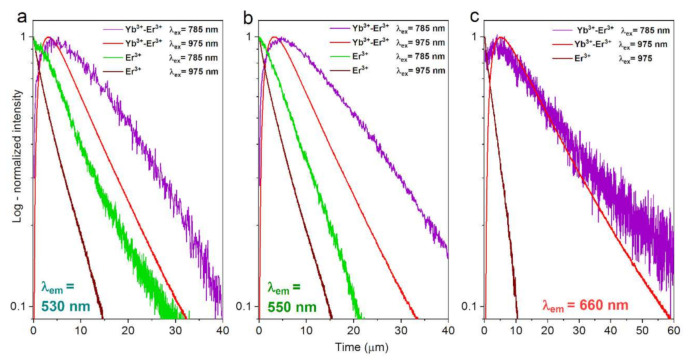
Normalized UC emission decay curves for the YVO_4_: Er^3+^ and YVO_4_: Yb^3+^-Er^3+^ nanomaterials, excited at λ_ex_ = 785 or 975 nm; monitored at λ_em_ = 530 (**a**), 550 (**b**) and 660 nm (**c**).

In order to confirm the validity of the postulated UC emission mechanisms, we measured the UC emission decay curves for the YVO_4_: Er^3+^ and YVO_4_: Yb^3+^-Er^3+^ nanomaterials, excited at λ_ex_ = 785 or 975 nm; monitored at λ_em_ = 530 (a), 550 (b) and 660 nm (c), which are presented in Figure 6. It is clear that, in the case of the samples co-doped with Yb^3+^-Er^3+^ ions (violet and red curves), the ETU mechanism dominates in the UC processes, which is manifested by the appearance of the rise curve in the initial parts of the curve profiles (with maximum intensity around ≈3–4 µs).

Whereas, in the case of the single-doped samples, containing only Er^3+^ ions, we observe only simple decay profiles, without any rise component, alike at 975 and 785 nm excitations, confirming the dominant contribution of the GSA and ESA mechanisms. Note, the rise curves/components, which are typically observed in many up-converting materials, are related to the energy transfer from the sensitizer (light harvesting ion) to the emitting ions, which, in our case, are Yb^3+^ and Er^3+^ ions, respectively.

The deviations from the pure exponential character of the recorded luminescence decay curves are mainly due to the quenching effects, such as interionic cross-relaxation processes. That is why we simply used the following equation to estimate the average UC emission lifetimes for all observed transitions in the studied systems:

(4) $$\tau =\frac{\int I\left(t\right)\;\cdot \;tdt}{\int I\left(t\right)dt}$$

where *τ* is the average decay time of UC luminescence and *I(t)* is the intensity at time *t*. The calculated lifetime values are given in Table 3. As expected, in the case of the Yb^3+^-Er^3+^ co-doped systems the UC lifetimes are much longer (≈11–24 µs) compared to the ones doped only with Er^3+^ (≈3–8 µs).

This is simply due to the presence of Yb^3+^ in the first case (the intrinsic lifetime of the Yb^3+ 2^F_5/2_ excited state), and the related energy transfer to the emitting Er^3+^ ions, leading to the overall prolongation of the lifetimes. On the other hand, the UC lifetimes are almost twice as long for the systems excited with 785 nm laser (higher energy), compared to the 975 nm excitation. This is plausibly due to the excitation of the electrons to higher excited states (^4^G_11/2_) with a 785 nm laser (see Figure 5), subsequently leading to the longer relaxation time (via more intermediate excited states) to the emitting levels.

**Figure 7:**
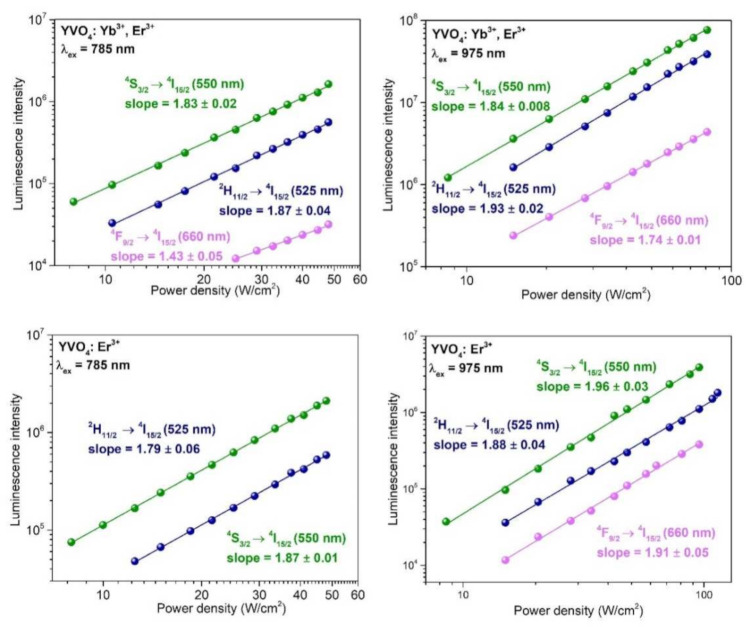
The log–log plots showing the dependences of the integrated UC luminescence intensity on the laser power for the samples YVO_4_: Er^3+^ and YVO_4_: Yb^3+^-Er^3+^. The determined slope values (by linear fitting) correspond to the number of photons participating in the particular transitions.

In order to determined and confirm the number of photons required for UC processes in the systems studied, the dependences of the integrated luminescence intensity (for each emission band) on the applied laser power were investigated and are presented as log–log plots in Figure 7. As expected, all emission bands of Er^3+^ were associated with two-photon transitions, both for λ_ex_ = 785 nm and λ_ex_ = 975 nm, alike for the single- and co-doped nanomaterials, as evidenced by the determined slope values, which are significantly higher than unity (one-photon process), being typically close to the ideal value of two (two-photon process).

The observed deviations from the ideal value (2) are common to UC materials, and they are typically associated with the processes of non-radiative quenching of the excited states of Er^3+^, such as multi-phonon relaxation and cross-relaxation phenomena [9]. To determine the number of photons participating in the transitions associated with the observed UC emission bands, we used the well-known relation *I_UC_* ∝ (*I_pump_*)*^n^*. In this relation, *I_UC_* is the UC emission intensity, *I_pump_* is the pump laser power density, and n is the number of photons involved in the UC mechanism. Performing a simple linear fitting, *n* can be calculated from the slopes of the plotted UC emission intensity as a function of the pump power in both the logarithmic representations [89,90,91].

## 4. Conclusions

Here, we demonstrated the possibility of generating pure green UC emission by changing the UC mechanisms via manipulating the excitation wavelengths and the elemental composition of the dopants, thereby, resulting in altered energy migration pathways and ET processes. We achieved this goal by suppressing the population of the ^4^F_9/2_ level of Er^3+^ in inorganic, vanadate-based up-converting nanoparticles.

The nanomaterials of interest were single- or co-doped YVO_4_: Er^3+^ and YVO_4_: Yb^3+^-Er^3+^, respectively, obtained by a combination of hydrothermal and calcination methods. The synthesized compounds showed intense, visible to the naked eye green UC luminescence that was observable at various excitation wavelengths, i.e., λ_ex_ = 785 nm or λ_ex_ = 975 nm. The influence of the excitation wavelength and elemental composition on the intensity of the UC emission was investigated.

This study showed that, in order to obtain pure green UC emission (without any contribution of the red emission band in the spectrum) from the Er^3+^-doped inorganic matrices, the optically active phase should not contain Yb^3+^ ions and should be excited with higher-energy NIR light, such as a 785 nm laser, instead of the commonly used 975/980 nm lasers. The proposed strategy might be particularly important from the point of view of optoelectronics, lighting techniques, energy conversion etc.—in other words, whenever it is desired to generate light with a pure, single color.

## Figures and Tables

**Table 1:** The values of color coordinates, color purity and CCT for the synthesized YVO_4_: Er^3+^ and YVO_4_: Yb^3+^ and Er^3+^ nanomaterials.

Dopants	λ_ex_ (nm)	Color Coordinates				Color Purity (%)	CCT (K)
		*x*	*y*	*x_d_*	*y_d_*		
Er^3+^	785	0.2845	0.7022	0.2841	0.7066	98.9	6179
Er^3+^	975	0.2937	0.6943	0.2934	0.6997	98.6	6055
Yb^3+^, Er^3+^	785	0.2965	0.6901	0.2966	0.6959	98.5	6018
Yb^3+^, Er^3+^	975	0.2731	0.7101	0.2722	0.7186	97.9	6334

**Table 2:** Comparison of spectral characteristics of different Er^3+^-activated luminescent materials, based on the vanadate matrices.

Host	Dopant Ions	Detectable Red Emission Band	λ_ex_ (nm)	Excitation in the I-BW	Refs.
YVO_4_	Er^3+^	No	785	Yes	This work
	Er^3+^	Yes	975	No	
	Yb^3+^-Er^3+^	Yes	785	Yes	
	Yb^3+^-Er^3+^	Yes	975	No	
YVO_4_	Er^3+^	Yes	305–340	No	[73]
	Yb^3+^-Er^3+^	Yes	290–330	No	
YVO_4_	Er^3+^	No	223	No	[74]
YVO_4_	Er^3+^	Yes	310	No	[75]
YVO_4_	Er^3+^	No	300	No	[76]
YVO_4_	Er^3+^	No	317	No	[77]
YVO_4_	Yb^3+^-Er^3+^	Yes	980	No	[51]
YVO_4_	Yb^3+^-Er^3+^	Yes	975	No	[57]
YVO_4_	Yb^3+^-Er^3+^	Yes	980	No	[78]
YVO_4_	Yb^3+^-Er^3+^	Yes	985	No	[31]
YVO_4_	Yb^3+^-Er^3+^	Yes	970	No	[79]
YVO_4_	Yb^3+^-Er^3+^	Yes	980	No	[54]
YVO_4_	Yb^3+^-Er^3+^	Yes	980	No	[80]
YVO_4_	Yb^3+^-Er^3+^	Yes	980	No	[28]
YVO_4_	Yb^3+^-Er^3+^	Yes	980	No	[81]
YVO_4_	Yb^3+^-Er^3+^	No	257	No	[82]
	Yb^3+^-Er^3+^	Yes	980	No	
Ba_2_GdV_3_O_11_	Yb^3+^-Er^3+^	Yes	978	No	[62]
K_3_Y(VO_4_)_2_	Yb^3+^-Er^3+^	Yes	980	No	[60]
GdVO_4_	Yb^3+^-Er^3+^	Yes	980	No	[83]

**Table 3:** Determined average UC emission lifetimes for the YVO_4_: Er^3+^ and YVO_4_: Yb^3+^-Er^3+^ nanomaterials (excited at λ_ex_ = 785 or 975 nm) for the transitions ^2^H_11/2_⟶^4^I_15/2_, ^4^S_3/2_⟶^4^I_15/2_ and ^4^F_9/2_⟶^4^I_15/2_.

Dopant Ions (λ Excitation)	UC Luminescence Lifetimes (τ) for the Transitions		
	^2^H_11/2_⟶^4^I_15/2_ (530 nm)	^4^S_3/2_⟶^4^I_15/2_ (550 nm)	^4^F_9/2_⟶^4^I_15/2_ (660 nm)
Yb^3+^-Er^3+^ (975 nm)	11.35 µs	11.66 µs	19.38 µs
Yb^3+^-Er^3+^ (785 nm)	20.12 µs	20.76 µs	24.26 µs
Er^3+^ (975 nm)	3.37 µs	3.58 µs	3.05 µs
Er^3+^ (785 nm)	7.73 µs	7.01 µs	-

## Data Availability

All of the relevant data are available from the correspondence authors upon reasonable request. Source data are provided with this paper.
